# Supplementary material for: Are carbon emissions trading and green financial instruments synergistic? -Comprehensive quantitative research based on content analysis
Source: PLoS One. 2024 Mar 7;19(3):e0298601. doi: 10.1371/journal.pone.0298601 (PMC10919601; doi:10.1371/journal.pone.0298601)
Supplement: S1 Data — (ZIP) [file pone.0298601.s001.zip › Data/地级市Z_F工作报告.zip!/─┌├╔╣┼/═¿┴╔╩╨/└┤╫╘╣1⁄2╓┌║┼í╛┬φ┐╦╩2╛▌═°í┐_2017.docx]

各位代表：

现在，我代表市人民政府向大会报告工作，请予审议，并请市政协委员和列席会议的同志们提出意见。

一、2016年工作回顾

过去一年，在市委的坚强领导下，在市人大、市政协的大力支持下，市政府认真贯彻执行中央和自治区各项决策部署，全面落实新发展理念，着力推进供给侧结构性改革，统筹做好稳增长、促改革、调结构、惠民生、防风险各项工作，经济社会发展取得新进步，实现了“十三五”良好开局。

经济运行总体平稳。地区生产总值完成1949.4亿元，增长7.4%；限额以上固定资产投资完成1475.5亿元，增长14.5%；社会消费品零售总额完成515.3亿元，增长9.8%；一般公共预算收入完成128.3亿元，增长6.5%；城镇常住居民人均可支配收入达到27444元、增长8.2%，农村牧区常住居民人均可支配收入达到11585元、增长7.7%。先行指标稳中向好，工业用电量增速全年保持20%以上，在东北经济区处于领先地位；铁路货物发送量平稳回升，达到4740万吨；新增社会融资总量405亿元，增加77亿元，保障了经济平稳运行。

“三去一降一补”工作扎实推进。压减煤炭产能近1000万吨。出台中心城区阶段性购房补贴政策，设立“房产超市”，全市减少商品房库存230万平方米。金融机构不良贷款余额和不良贷款率实现“双降”，置换政府存量债务149.2亿元。实施补短板9大类、43项重点工程。全面落实“营改增”等减税降费政策，为企业减负10亿元以上。出台企业降本增效30条意见，助力企业解难脱困。争取大用户直购电量47.3亿度，减少企业电费支出7.2亿元。通过“助保贷”为117户中小企业发放贷款10亿元。全年累计降低企业生产成本20.2亿元，规模以上企业利润总额增长15%以上。我市企业减负增效工作获得国务院第三次大督查通报表扬。

产业转型升级步伐加快。产业结构进一步优化，三次产业比调整到13.4：50.2：36.4。农牧业结构稳中调优，压减籽粒玉米311万亩，粮改饲267万亩，农产品“三品一标”认证面积251万亩，粮食总产达到139.2亿斤；突出发展肉牛产业，新增优质肉牛24万头、总量达到326万头，“科尔沁牛”获得农产品地理标志认证。新型工业化进程提速，实施500万元以上工业重点项目303个，完成工业固定资产投资688亿元，规模以上工业增加值增长9.3%。依靠科技创新，铝新材料、玉米生物技术、绿色农畜产品加工等优势主导产业向全产业链、中高端方向发展。成立内蒙古蒙东铝及铝加工产业技术创新战略联盟，铝新材料产业新增产品100种、优势产能近百万吨；玉米生物技术产业新增精深加工产品50个，玉米综合转化能力达到505万吨；绿色农畜产品加工企业发展到160家，农畜产品综合加工转化率达到61%。战略性新兴产业向规模化迈进，伯恩露笑蓝宝石晶体产量接近翻番，风电、光伏等新能源装机占总装机的40%。蒙医药产业规模化、市场化进程加快，蒙中药材基地达到7万亩，蒙成药产量占全国的50%以上。现代服务业快速发展，科尔沁500公里风景大道自驾游服务系统建设初具规模，自驾游占旅游总人数的60%，“东北看草原，自驾游通辽”品牌知名度正在提高；与国开行等金融机构签署了总规模2830亿元的金融合作框架协议，争取国家专项建设基金32亿元；开通“通满欧”跨境货运班列和大连港集装箱班列，举办中蒙博览会通辽国际商品交易会暨中蒙“3+3”区域合作会议，物流公共服务平台建设取得新进展；万达广场、长春欧亚商业综合体投入运营；电子商务交易额成倍增长，通辽电子商务产业园获国家“2016十佳电子商务园区”殊荣。

基础保障能力不断提升。通辽至京沈高铁新民北站铁路、扎鲁特至青州±800千伏特高压直流输电工程和通辽至鲁北、大沁他拉至白家湾子高速公路项目全面开工，霍林河至阿力得尔一级公路、舍伯吐至大兴二级公路建成通车，通霍、通大等铁路电气化改造工程顺利实施，霍林河民航机场试飞成功。启动北部1000平方公里区域战略环评，煤电铝烟气污染物趋零排放示范工程开工建设，区域燃煤锅炉治理任务超额完成，中央环保督察交办和反馈问题整改见到成效。完成科尔沁沙地“双千万亩”综合治理363万亩、重点区域绿化26.5万亩，荣获“全国绿化模范城市”称号。

发展活力进一步增强。行政许可事项由61项压减到41项，审批时限缩减64%，公布了中介服务、公共服务事项、政府权责“三张清单”。推广“煤电铝”一体化循环经济发展模式，电力洼地效应进一步显现，霍林河循环经济示范工程荣获“国家优质工程奖”。组建水务、交通等4家投融资平台，通过设立“美丽乡村”产业基金等方式多渠道融资200多亿元。商事制度改革和大众创业、万众创新政策深入实施，市场主体新增2.9万户、总量突破20万户，被国家确定为东北地区民营经济发展改革示范城市。农村牧区综合改革取得新进展。对外贸易平稳增长，进出口总额超过4亿美元。与保定市缔结友好城市，与吉林大学等高校开展合作。人才强市工程深入推进，引进急需高层次人才138名。实施精准招商，新签约千万元以上项目177个，实现到位资金685亿元。

民生状况显著改善。各级财政民生支出280亿元，占一般公共预算支出的78%。全力推进脱贫攻坚，4万贫困人口稳定脱贫，科尔沁区、开发区、霍林郭勒市率先解决区域性整体贫困。城镇新增就业1.8万人，农牧民转移就业47万人，城镇登记失业率控制在3.7%。城乡低保、特困救助供养人员保障标准和补助水平稳步提升，建成农村牧区互助养老幸福院80所，新增养老床位8529张。农村牧区“十个全覆盖” 工程基本完成。城镇基础设施和公共服务进一步完善，辽河公园荣获“中国人居环境范例奖”。百姓安居、供水改造和水质提升、贫困人口免费体检、城市公共交通等十项民生工程取得明显成效。国家卫生城市、全国文明城市创建取得阶段性成果，凝聚了全市广大干部群众干事创业、建设美好通辽的巨大正能量。

各项社会事业协调发展。开展民族团结进步创建活动，各民族大团结的良好局面进一步巩固。公共文化服务标准化、均等化建设走在自治区前列，市图书馆新馆主体完工，乌力格尔艺术馆和版画艺术中心基本建成，哈民考古遗址公园向公众开放。成立全区首家科技银行，我市被科技部认定为国家农业科技园区。组建市教育教学研究院，完成农村牧区338所学校教师周转宿舍和21所蒙古族小学“大通铺”改造工程，霍林郭勒市、库伦旗通过国家义务教育基本均衡县评估认定，自治区全面提升基础教育办学水平现场推进会在我市召开。足球改革发展初见成效，承办了自治区高水平联赛，获得蒙超、蒙甲联赛冠军。城市公立医院综合改革全面推开，市医院晋升三级甲等综合医院，旗县综合医院全部晋升二级甲等医院，第二轮京通医疗卫生合作全面启动，举办了内蒙古第二届国际蒙医药博览会，荣获“中国蒙医药之都” “全国无偿献血先进市”和“全国基层蒙中医药工作先进市”称号。统一权威的食品药品监管体系基本建立，监管能力进一步提高。城乡居民基本医疗保险整合工作全面完成，实现城乡医疗保险一网化运行管理。平安通辽建设扎实推进，被评为“全区普法依法治理先进市”。“双拥”优抚政策有效落实，连续四届获 “全国双拥模范城”称号。人民防空、地震气象、广播电视、档案史志、宗教事务、外事侨务等工作取得新成效，红十字、妇女儿童、老龄和残疾人等事业实现新进展。

政府自身建设得到加强。扎实开展“两学一做”学习教育，持续推进作风建设，公务员队伍素质得到提升。认真执行人大及其常委会的各项决议、决定，自觉接受政协民主监督，主动接受社会和舆论监督，认真办理市人大代表建议和政协委员提案。全面落实从严治党主体责任和党风廉政建设“一岗双责”，加强审计监督，规范行政权力运行。建立政府文件合法性审查机制，大力推进政务公开，完善新闻发布制度，推广电子政务和网上办事，行政效能进一步提高，依法行政工作进入全区前列。

各位代表，成绩来之不易，这是自治区党委、政府和市委总揽全局、正确领导的结果，是市人大和市政协认真监督、大力支持的结果，是全市各族干部群众守望相助、共同奋斗的结果。在此，我代表市政府，向全市各族干部群众，向所有关心支持通辽建设与发展的同志们、朋友们，表示衷心的感谢并致以崇高的敬意！

在看到成绩的同时，我们也清醒认识到，发展中还存在一些不容忽视的问题：我市经济结构不优，产业层次不高，综合实力不强，人均地区生产总值低于全区平均水平，欠发达的基本市情没有根本改变；稳增长压力较大，部分企业生产经营困难，民间投资增长势头有所放缓，民营经济发展不够充分；现代服务业和战略性新兴产业发展不足，具有市场竞争力的现代产业体系尚未形成；依靠改革创新促进动能转换的体制机制尚未建立，对外合作交流水平有待提高，全方位开放力度仍需加大；财政收入质量不高，非税收入比重较大，民生和社会事业刚性支出增加，收支矛盾突出；城乡居民收入水平相对较低，脱贫攻坚任务十分艰巨；生态环境脆弱，水资源、环境容量等约束趋紧，绿色发展任重道远；政府依法行政能力和服务效率有待提高，引领经济发展新常态的创新能力有待增强，社会治理现代化水平还需提升。市政府作为“作战部”，在困难和挑战面前，我们必须直面问题不回避、敢于担责不推托，牢记使命、坚定信心、主动作为，团结带领广大干部群众，多方争取社会各界鼎力支持，在实干中把握机遇，在实干中破解难题，在实干中谋求发展，决不辜负全市各族人民的期望和重托！

二、2017年工作安排

今年是实施“十三五”规划的重要一年，是我市加快融入东北振兴、实现跨越发展的关键一年。做好政府工作，必须紧密团结在以习近平同志为核心的党中央周围，全面贯彻党的十八大和十八届三中、四中、五中、六中全会精神，深入贯彻习近平总书记系列重要讲话精神和治国理政新理念新思想新战略，全面落实自治区第十次党代会、自治区党委十届二次全会、自治区十二届人大六次会议和市第五次党代会、市委五届三次全会作出的重要部署，统筹推进“五位一体”总体布局，协调推进“四个全面”战略布局，坚持稳中求进工作总基调，践行新发展理念，以提高发展质量和效益为中心，以推进供给侧结构性改革为主线，坚守“三条底线”，促进“五化”协同，建设“六大产业基地”，实施“七大工程”，调优一产、做强二产、加快三产，做好基础设施、产业发展、生态环保、脱贫攻坚、城乡统筹、社会事业和民生改善等各项重点工作，以优异成绩迎接党的十九大胜利召开，向自治区成立70周年献礼。

主要预期目标是：地区生产总值增长8%；限额以上固定资产投资增长15%以上；社会消费品零售总额增长10%；更加注重财政收入质量，税收收入同口径增长9%以上；城乡常住居民人均可支配收入分别增长9%和10%。全面完成自治区下达的各项约束性指标。

实现上述目标，我们要牢牢把握以下基本要求：

率先跨越发展。这是自治区推进新一轮东北振兴战略对蒙东地区提出的最新定位。要对接东北、引领蒙东，强化创新驱动、改革推动、开放带动，经济增速高于全区平均水平，加快打造自治区重要增长极。

严守生态底线。这是实现通辽转型发展的基本保障。要坚持美丽与发展双赢，决不以牺牲环境、浪费资源为代价换取一时的经济增长，既要金山银山，更要绿水青山，推动绿色强市、绿色惠民，形成人与自然和谐发展新格局。

坚持民生为先。这是做好一切工作的根本所在。要始终坚持以人民为中心的发展思想，扎实做好普惠性、基础性民生建设工作，坚决打赢脱贫攻坚战，在全面建成小康路上决不让一个困难群众掉队。

确保安全稳定。这是建设各族人民和谐幸福美好家园的重要基础。没有安全稳定，一切发展都归零。要把安全稳定作为发展的“压舱石”，用法治精神凝聚改革共识、规范发展行为、促进矛盾化解、维护公平正义，为各族人民幸福生活创造和谐环境。

今年，要重点做好以下九个方面的工作：

（一）保持经济平稳健康运行

加快投资拉动、企业行动、创新驱动，以中高速增长赢得发展先机。

强化基础设施项目投资。以构建铁路、公路、航空、市政、能源、信息通信和水利七大网络体系为重点，扩大有效投资，打造跨越发展新支撑，全年完成限额以上固定资产投资1700亿元以上。水利网方面，突出“七网融合、水网当先”，实施“引水节水建设工程”。坚持“外引”与“内蓄”并举，推进“引绰济辽” “辽西北供水”通辽支线等重大跨流域引水调水工程前期工作。开工建设嘎海山水库，毛都水库竣工蓄水。继续抓好山区水土保持和农村牧区中小水利工程建设，完成120个嘎查村饮水安全巩固提升工程。把农业节水作为方向性、战略性大事来抓，大规模实施农业节水工程，普及高标准管灌，推行浅埋滴灌，年节水能力达到6亿立方米以上。铁路网方面，完成通辽至京沈高铁新民北站铁路和京通、通让等铁路电气化改造主体工程，集通铁路电气化改造项目开工建设，通霍铁路尽快提速运行。公路网方面，规划启动县县通高速公路工程，建成通辽至鲁北高速公路，续建大沁他拉至白家湾子高速公路、舍伯吐至哈根庙一级公路、鲁北至开鲁二级公路，实施大广高速奈曼东连接线一级公路改造工程，开工建设金宝屯至甘旗卡、大林至康平、太平川至宝龙山二级公路。继续实施公路畅通工程。改造全市公铁平交道口。新建通村公路663公里，较国家计划提前三年实现行政嘎查村全部通沥青（水泥）路。航空网方面，改扩建通辽机场，通航运营霍林河民航机场，开工建设奈曼、库伦通用机场。市政网方面，规划建设中心城区中水回用主管网工程，推动沿线区域中水回用。提高中心城区供水能力，扩建新城区通新水厂，开工建设三义堂水源地工程，铺设供水管道26公里，强化居民供水二次管网统一管理。继续实施水质提升和排水管网建设改造工程。加快实现中心城区热网互联互通，建设供热、燃气一体化平台。积极推进地下综合管廊工程。能源网方面，加快建设扎鲁特至青州±800千伏特高压直流输电工程，实施通辽至霍林郭勒天然气管道项目，推进扎哈淖尔多能互补集成优化示范项目建设，对农村电网进行新一轮升级改造。信息通信网方面，坚持规划先行、资源共享、分步实施的原则，建设智慧城市，推进“三网融合”，推动“宽带中国”示范城市建设，用信息化引领经济社会跨越发展。

大力发展非公有制经济。全面落实国家、自治区鼓励和支持非公有制经济加快发展的各项政策措施，坚决扫除各种思想障碍和体制障碍，让非公有制经济扛大梁、挑重担、作贡献。构建新型政商关系，把干部服务企业的行为转变成组织发展市场主体的机制，建立于公有利、于己无忧的制度体系。市旗两级领导包联服务优秀企业，为企业家营造安心经营、放心投资的宽松环境。着力振兴实体经济，实施 “企业和企业家培育工程”，强化金融信贷、科技创新、人才培养、生产要素配置等政策支持，培育一批骨干龙头企业、创新型中小企业，促进本土企业与外来企业公平竞争、大中小企业竞相发展。因企施策、一企一策，力促生产企业达产达效。引导企业发扬“工匠”精神，开展质量提升行动，发挥市长质量奖激励作用，促进企业开展质量体系认证。实施品牌战略，每个旗县新增1枚以上著名商标，支持库伦蒙药等企业争创驰名商标。

实施创新驱动战略。发挥科技创新引领作用，加快建立以市场为导向、企业为主体、产学研用相结合的科技成果转化推广体系。完善科技奖励和补助政策，鼓励科技人员创新创业，支持企业应用高新技术。加强蒙医药国家级重点实验室等创新平台建设，在铝新材料、蒙医药等重点领域开展技术攻关，培育申报高新技术企业。积极创建自治区级高新技术产业开发区。深化与内蒙古民族大学等区内院校和科研院所合作，加强与清华大学、吉林大学、中国农业大学、东北大学等高等院校和科研院所的战略合作。推动大众创业、万众创新，培育各级各类创业孵化器和众创空间，建设标准化创业孵化园区，支持发展“互联网+”创业园，建设家庭服务业公共服务平台，培育创新文化，释放全社会创新创业活力。

（二）深入推进供给侧结构性改革

持续加大“三去一降一补”工作力度，努力实现供求关系新的动态平衡，全面提高经济发展的质量和效益。

稳步有序去产能。严格执行环保、能耗、质量、安全等相关法律法规和标准，鼓励企业兼并重组，化解低端落后产能，为先进产能和有效供给释放空间，完成自治区下达的煤炭去产能任务，原铝就地转化率达到80%以上，酸、胶、药等玉米精深加工产能达到300万吨。

多措并举去库存。综合运用土地、金融等政策，有效释放刚性需求和改善性需求，加大对城镇居民首套住房、农牧民进城购房信贷资金供给，扩大住房消费。发挥棚改去库存主渠道作用，推进棚改货币化安置。加强房地产供给侧管理，培育住房租赁市场，鼓励在建开发项目与文化、体育、养老等业态融合，促进房地产市场平稳健康发展。

积极有效去杠杆。创新投融资方式，加快发展多层次资本市场，支持企业在新三板、区域性股权交易市场挂牌，推动优质企业上市融资。跟进落实与金融机构签署的战略合作协议。设立政府引导性投资基金，在基础设施、现代物流、科技创新、文化旅游、肉牛产业等基金的设立运作和管理上取得实质性进展。推广政府与社会资本合作模式，做大做强融资平台，稳步提升融资能力。强化政府债务管理，规范政府举债行为。

千方百计降成本。落实国家、自治区税费减免政策，减少审批事项，加大涉企收费清理力度，规范中介服务收费，持续开放“绿色通道”，降低企业制度性交易和用工、物流等成本。全力争取大用户直购电交易电量指标，积极拓展局域微网、电价补贴覆盖面。完善“助保贷”合作机制，落实中小企业贷款10亿元以上。设立转续贷基金，解决企业短期融资难问题。

精准加力补短板。从制约经济社会发展的重要领域和关键环节、从人民群众迫切需要解决的突出问题着手，深入实施补短板工程，努力在基础设施、脱贫攻坚、现代服务业、生态环境和民生保障等领域取得突破，提高通辽发展的“木桶容量”，形成新的增长动能。

（三）推动工业提质增效

新型工业化是推进“产业转型升级工程”的主体。突出工业主导地位，调整存量、优化增量、提升质量，推动工业向高端化、智能化、绿色化、服务化方向发展。全年实施500万元以上工业重点项目330个，完成投资768亿元，同比增长13%以上。规模以上工业增加值增长9.5%。

改造升级传统产业。运用新技术新业态新模式改造提升传统产业。构建“煤电铝”一体化竞争新优势，大力推进产能置换、市场合作、产品创新，重点抓好麦达斯高端铝材、奇瑞汽车铝轮毂及配件、忠大铝业航空级铝厚板、联晟双零箔等铝精深加工项目，推动铝后加工向高端产品延伸，打造绿色循环、技术先进、具有市场话语权的千亿级产业集群，建设铝新材料等有色金属产业基地。突出玉米全株全产业链建设，加快玉米生物技术产业向食品、医药级产品制造转变，重点推进梅花生物、华曙生物、黄河龙生物等企业延伸产业链条，建设国家重要的玉米生物技术产业基地。推动绿色农畜产品加工业向高端化供给转变，变“粮仓肉库”为“精品超市”。支持企业进行信息化集成应用与融合创新，大力发展智能制造、个性化生产，打造传统工业升级版。

做大做强战略性新兴产业。抢抓自治区高标准建设国家大数据综合试验区的机遇，充分利用国家网络规划二级节点城市的优势，加快发展大数据产业，面向东北打造区域性大数据产业园和云计算应用中心。积极发展清洁能源产业，重点推进北部区域微电网和电力外送通道建设，全力争取特高压输电份额，打造区域清洁能源输出基地。实施“蒙医蒙药发展工程” ，加强蒙医药基础建设和研究，建立中国蒙药基因中心、信息中心、检测中心，积极申建内蒙古蒙医药大学。提升蒙中药材种植、生产加工、技术研发、医疗应用规模和水平，打造具有良好市场前景的蒙医药拳头产品，建设全国蒙医药产业发展核心区和示范区。办好内蒙古第三届国际蒙医药博览会，加强国际国内交流合作，增强“中国蒙医药之都”的权威性和影响力。推进福耀集团新建2条汽车玻璃生产线，扩大伯恩露笑蓝宝石晶体生产规模，加快京蒙高科干细胞项目产业化进程，培育形成新的经济增长点。

推进工业园区集约集聚发展。完善工业园区发展规划，优化产业布局和功能区划分，促进园区差异化、特色化发展。加大旗县工业园区道路、供水、供气、通讯、污水处理等基础设施建设力度，配套建设电源点，强化电力保障，提高承载力和吸引力。进一步理顺管理体制机制，以旗县为单位整合同城园区，规范运行管理，规划启动科尔沁工业园区东区搬迁改造。创新资源配置方式，推动园区跨区域一体化发展，实现资源配置效益最大化和效率最优化，提高园区投资强度和产出强度。

（四）促进农牧业现代化

农牧业现代化是推进“产业转型升级工程”的基础。顺应食品消费结构升级趋势，培育“绿色科尔沁、健康全人类”的生态农牧业发展理念，加快农牧业供给侧结构性改革，提高农牧业综合效益和竞争力。

增强农牧业可持续发展能力。进一步优化耕地功能布局，大力发展生态节水型农牧业，推广良种良法良机集成配套和水肥一体化模式，新增低压管灌和浅埋滴灌节水灌溉面积50万亩以上，加快建成1000万亩节水高产高效粮食功能区，粮食总产稳定在140亿斤以上。加强草牧场保护和饲草基地建设，粗饲料生产能力达到460亿斤，牲畜存栏稳定在2200万头只以上，肉类产量70万吨以上。完善农牧业社会化服务体系，统防统治玉米螟，提高重大疫病防控能力。推广玉米生产全程机械化，农牧业综合机械化率达到85%以上。大力培育新型农牧业经营主体，发展多种形式的适度规模经营，促进农牧业节本增效。推行农牧业清洁生产方式，深入开展化肥农药零增长行动，发展绿色种植、循环种养、休耕轮作，实现农牧业绿色可持续健康发展。

扩大农畜产品有效供给。调优种植业，推进粮改饲、粮改经，青贮玉米发展到500万亩，红干椒、蒙中药材等经济作物达到300万亩。引导多品种玉米种植，继续压减籽粒玉米70万亩，将玉米生产面积控制在1250万亩。因地制宜发展设施农业，稳步提高“菜篮子”自给能力。画好“五牛图”，突出发展肉牛产业，加快“肉牛大市”向“肉牛强市”转变。力促科尔沁牛业、邦杰肉牛、谷润肉业达产达效，推进伊赛肉牛、牧合家肉牛产业化项目建成投产，全市肉牛实际加工突破20万头。重点培育科左中旗成峰、开鲁县雨田、科左后旗浩远等7个肉牛交易市场，全市交易量达到160万头以上。实施肉牛种子工程，实现良种成为产品、繁育成为产业。扶持壮大科尔沁肉牛种业基地，引进和培育优质肉牛品种，加快本地肉牛品种改良，种牛规模达到700头。以母牛扩繁“万千百十”示范工程为重点，推进规模化、标准化养殖。以小规模、大群体方式，壮大西门塔尔肉牛养殖规模，以专业合作社和龙头企业为主体，稳步扩大安格斯等高端肉牛养殖规模，肉牛存栏达到350万头。打好绿色牌，发挥生态农牧业资源优势，做优做精科尔沁牛、库伦荞麦、开鲁红干椒、奈曼蒙中药材、科左后旗水稻、扎鲁特旗杂粮杂豆等一批绿色优质农畜产品，提升品牌影响力。加强农产品质量安全监管，开展特色农畜产品标准化生产示范，提升农产品供给质量，“三品一标”认证面积达到300万亩，产品产量达到150万吨，加快建成绿色农畜产品生产加工输出基地。

拓展农牧业产业链价值链。坚持用工业化思维、市场化理念、信息化手段改造提升农牧业，推动一二三产业融合发展。壮大农牧业产业化龙头企业，完善龙头企业与农牧民利益联结机制，建立种养加一条龙、产供销一体化的生产经营体系，带动35万农牧户增收致富，农牧民来自产业化收入的比重达到50%。完善农畜产品市场销售体系，建设区域性农畜产品批发市场，构建体验店、展示直销店、网店等全方位立体直销模式，搞活“农博”经济，加快形成买全区卖全国的农畜产品流通大格局。挖掘农牧业的多功能性，发展“互联网+”农牧业，尽快催生农村牧区新产业新业态。

（五）加快发展现代服务业

现代服务业是推进“产业转型升级工程”的重点。加快服务业发展提速、比重提高、水平提升。

大力发展全域旅游。按照自治区建设国内外知名旅游目的地的决策部署，贯彻全区旅游发展大会精神，实施“全域旅游、四季旅游、旅游+”发展战略，把旅游业打造成新型战略支柱产业，把通辽打造成国内外知名的自驾游体验基地、度假休闲康养基地和高品质旅游城市。突出品牌化，发挥我市旅游资源“最字号”“源字号”“独字号”优势，高标准编制全域旅游发展规划，以科尔沁500公里风景大道建设为依托，出台加快旅游业发展的实施意见，坚持规划引领、市场运作、企业主体、社会参与，推动形成“资源有机整合、精品景区荟萃、产业深度融合、旅贸结合发展”的大开放旅游发展格局。突出多样化，依托我市草原、沙漠、森林、湖泊、湿地等多类型地貌，深度挖掘乌力格尔、安代、赛马、博克、版画等多元民族文化，差异化开发特色产品，标准化打造服务系统，“串珠成链”形成四季旅游组合优势。突出集成化，在北部草原建设高端体验式景区和民族风情园，围绕蒙古服饰、蒙古音乐、蒙古建筑及民俗风情等搞汇集、造精品、做品牌，全力打造自治区蒙古风情、蒙古文化、蒙古生活的“锦绣中华”，实现一个景区浓缩多种文化。大力发展民族特色餐饮，提高“中国蒙餐之都”影响力。突出主题化，促进旅游与文化、体育、农牧业、扶贫、健康养老等行业的深度融合。加强旅游区域化合作，推动建设霍林郭勒—乌拉盖旅游集聚区。以完善自驾游服务系统为重点，建设精品旅游线路。南部以蒙医康养体验游、沙漠瀚海探险游、科尔沁文化探秘游为主，打造蒙医康养之旅。北部以民俗风情体验游、原始草原生态游、科尔沁文化体验游为主，打造游牧体验之旅。创新旅游推介模式，大力发展智慧旅游，组织开展“2017通辽文化旅游季”等系列活动，全面提升科尔沁旅游产品的吸引力和竞争力。

构建现代商贸物流体系。优化中心城区商业网点布局，重点打造老城区长春欧亚、五洲国际、万达广场和新城区日升日美、万力城、金都王府井6大商业集聚区。支持旗县城关镇建设一批特色商业区，推进社区商业“双进工程”，打造社区商业便民服务圈。推动铁路、公路、航空物流无缝对接，把枢纽优势转化为发展优势，将我市打造成中蒙俄经济走廊重要支点、东北区域现代综合物流枢纽、蒙东区域性产业组织中心。优化中心城区物流业发展空间布局，重点推进开发区综合物流园区、科尔沁现代物流园区、空港物流园区、木里图工业物流园区和内蒙古东部煤炭交易中心建设。做大做强通辽内陆港等现有物流企业，积极引进国内外知名第三方物流企业，提升物流专业化、标准化、信息化水平，健全大宗物流、冷链物流、快递物流、国际物流、电子商务及配送体系。规划建设旗县特色物流园区、专业市场和物流配送中心，完善县乡村市场流通体系。加快航空口岸、保税物流中心、多式联运海关监管中心等海关监管场所申报建设，推进通满欧跨境货运班列常态化运行，畅通国际物流通道，形成“大进大出、优进优出”的大物流格局。

壮大金融服务业。制定出台扶持金融业发展的政策措施，鼓励域外商业银行在我市设立分支机构，支持驻市金融机构增设县域分支机构和电子服务网点，增强金融服务实体经济能力。农信社改制工作取得突破性进展，年内扎鲁特旗、库伦旗、科左后旗、开鲁县、霍林郭勒市农商行实现挂牌。全力推进科尔沁区农信社改制组建通辽农商行，加快构建股权结构更合理、体制机制更科学的现代法人治理结构。大力发展普惠金融，着力解决小微企业贷款难、农村牧区高利贷等问题。稳步推进农村牧区信用体系建设和农村小额担保基金试点工作。推动设立内蒙古股权交易东部运营中心、蒙东金融仲裁中心、通辽金融资产交易中心，搭建企业上市挂牌、融资平台。筹备设立地方性商业银行和民营银行，发展金融租赁、融资租赁等新业态，构建多层次、广覆盖的金融体系，新增社会融资总量450亿元以上，努力把通辽建成蒙东金融合作示范区。

积极培育新兴服务业。完善各级电子商务产业园综合服务功能，加快建设国家级电子商务进农村示范县项目，建成覆盖城乡的电子商务公共服务网络。引进和培育电商龙头企业，扶持优势特色电商平台，发展跨境电子商务和跨境电商体验店，推进传统企业线上线下融合，开辟实体经济发展新渠道。发展健康养老服务业，建设一批医养结合型养老机构、居家和社区养老服务中心等产业化项目，构建政府公共服务和社会化服务相结合、市场化运作的养老服务新模式。大力培育健身休闲、竞赛表演等体育产业，积极发展科技咨询、会展服务、教育培训、家政服务等产业，形成一批特色多样的新兴服务业态。

（六）统筹城乡区域协调发展

新型城镇化是区域整体实力和竞争力的集中体现。坚持规划引领、科学布局，以产城融合统筹城乡区域协调发展。

以人为本推进新型城镇化。严格执行《通辽市城市总体规划（2015—2030）》，编制控制性详细规划，打造“一核双心多点，一轴一带三区”的空间格局，加快建设区域性中心城市。做大做强中心城区，启动中心城区卫星城镇群规划编制，推动城市公共基础设施和公共服务向卫星城镇延伸下沉。加快城市更新改造，加强城市修补和生态修复。实施市政路桥工程，完成霍林河大街东段拓宽改造，开工建设新工二路、昆都仑南路、红光大桥等道路桥梁工程。实施园林景观工程，完成西拉木伦公园改造，实施辽河公园东延工程。实施重大公建工程，推进市医院新院区、蒙医中心医院、妇幼保健院建设，市图书馆新馆、科技馆等续建工程年内交付使用，规划建设高铁站前广场、新媒体传输总站和电视塔。市区两级联动，加快建国片区、城东片区征拆改造，积极解决中心城区棚户区改造历史遗留问题。优先发展城市公交，提高市民出行公共交通分担率。采取政府主导、市场化运作的方式，建设一批停车场、停车楼、物业配送中心等公共服务设施，完善社区功能，提升居住品质，建设宜居城市。建设魅力县城、特色小镇和美丽乡村，坚持规划先行、分步实施、重点突破，实施县城市容市貌、基础设施和公共服务集中攻坚，提升城关镇建设水平。全面启动苏木乡镇政府所在地综合改造升级工程。抓住科左中旗舍伯吐镇被列为第一批中国特色小镇的机遇，以肉牛产业和商贸物流为支撑，以民俗风情和史前文化为特色，打造融产业、旅游、文化、社区等功能于一体的特色小镇，引领带动全市特色镇建设。按照“市级奖补、旗县级为主、乡镇级为辅”的原则，建立农村牧区基础设施和公共服务投入长效机制，充分调动广大农牧民共建共管积极性，巩固新农村新牧区建设成果。深入推进农村牧区人居环境整治，建设既有现代文明、又具田园风光的美丽乡村。提升城镇管理水平，加大《通辽市城市市容和环境卫生管理条例》宣传和执法力度，提升城市法治化、精细化管理水平。持续推进“双城同创”，进一步理顺城市管理体制，明确市区两级城市管理职责和权限，组建城镇综合执法机构，提高城市综合治理能力。落实户籍制度改革措施，完善教育、医疗、社保、就业、住房保障等公共服务政策，促进农牧业转移人口市民化。

实施“三大区域”协同发展战略。制定出台霍扎区域一体化协同发展指导意见，编制一体化协同发展中长期规划纲要，推动霍林郭勒市和扎鲁特旗在制度创新、科技进步、产业升级、绿色发展等方面开展深度合作，建设北部区域改革创新试验区。以一体化的规划和一体化的管理，推进霍林郭勒工业园区和扎哈淖尔工业园区融合发展，实现园区产业布局、基础设施建设、矿产资源利用、生态环境保护、公共服务一体化，力争年内产值突破1000亿元，争创蒙东地区首个国家级高新技术产业开发区。推动科尔沁区、开发区和开鲁县优势互补、联动发展，突出中心城区生态和服务功能，形成高端产业和服务业聚集区。推动科左中旗、科左后旗、库伦旗和奈曼旗加快形成绿色经济产业带，培育壮大绿色种养业、有机食品加工业、生态旅游业，打造开放发展、绿色发展示范区。

（七）全面深化改革和扩大对外开放

改革出动力，开放出活力，通过大改革大开放促进跨越发展。

推进重点领域和关键环节改革。深化“放管服”改革，制定完善政府权责、公共服务事项、中介服务事项、涉企收费、随机抽查事项“五张清单”，全面实行清单管理制度，开展“减证便民”专项行动，方便群众办事创业。完善招投标业务流程和管理办法等规章制度，实行重点项目联合审批、上门服务，为创新创业者开辟绿色通道。深化电力体制改革，推动区域性电力体制综合改革示范区建设，加快建立售配电平台，支持用电企业参加直购电交易。推进国企国资改革，落实国企国资改革政策，激发国有企业活力。稳妥推进霍煤集团、通粮集团等国有企业改革。健全完善国资监管体制，实施国有资产集中统一监管，提高资源配置效率。深化商事制度改革，实行“多证合一”，加快推进工商登记全程电子化，积极探索简易注销登记，进一步放宽市场准入。推进农村牧区综合改革，深化农村牧区集体产权制度改革，有序推进土地草牧场所有权、承包权、经营权“三权分置”，稳妥开展土地确权颁证工作。积极推进供销社综合改革、农垦改革、农村水利水价改革。推进社会事业改革，抓好教育领域、医药卫生、食品药品监管和科技体制等各项重点改革。

实施对外开放引领工程。积极融入“一带一路”、向北开放战略，用好用活蒙古国“零关税”政策，尽快打通与蒙古国跨境旅贸直通线路，规划建设霍林河进口资源加工区和保税加工区，打造中蒙俄经济走廊重要节点城市。加快融入东北一体化发展，参与生产力布局调整和产业链重构，加强与东北经济区的能源输出合作，重点与长春一汽、忠旺铝材等企业对接，建设东北经济区清洁能源保障基地和装备制造业配套基地。规划实施一批基础设施互联互通工程，推进通辽至乌兰浩特至齐齐哈尔高速铁路、通辽至长春城际铁路等项目前期工作，争取更多项目列入新一轮东北振兴规划。主动融入京津冀协同发展战略，加强与华北铝业、顺鑫农业、盛世投资等企业对接，积极承接先进产能、项目、资本转移和科技、教育、医疗等资源外溢，建设京津冀产业转移集聚区。不断深化与港澳台地区交流合作，扩大同东南亚、欧美等国家的经贸往来，深度融入国内国际产业链、价值链、物流链，大范围、多层次承接人才流、资金流、科技流，在扩大开放中增创发展新优势，形成多边合作、八面来风的开放新格局。

加大招商引资引智力度。抢抓新一轮产业整合机遇，紧扣特色优势产业链，实施精准招商，以存量引增量，以资源引资金，以市场换合作。强化领导招商，落实领导干部招商责任，建立招商项目包联服务机制。强化专业招商，选派干部赴京津冀、环渤海、长三角、珠三角等地定点定向招商。强化产业链招商，围绕优势主导产业延伸升级和现代服务业开展招商，着力引进一批拉动作用强、发展潜力大的项目。年内新开工超亿元招商引资项目100个、实现到位资金700亿元以上。围绕人才短板，制定出台人才引进政策，坚持招商与招才并举、引资与引智联动，进一步拓展人才引进渠道，提高引进人才层次和待遇水平，推动人才与产业深度融合，打造高层次、高技能人才集聚地。

（八）加强生态建设和环境治理

绿色生态是通辽的潜力和希望。坚持绿色发展，扎实推进“生态环境保护工程”，坚守生态底线，不碰环保红线，让绿色扮美通辽。

加大生态保护和建设力度。坚持全域生态化、生态园林化、园林产业化，扩大生态建设投资，大规模开展国土绿化，完成科尔沁沙地“双千万亩”综合治理工程346万亩，实施重点区域绿化12万亩，大力推广使用乡土树种，提高城市园林绿化的近自然度，积极创建国家森林城市。进一步扩大科尔沁生态科技示范园建设规模，不断丰富内涵，加快建成蒙东地区大型生态科技观光示范基地。深化与蒙草抗旱等大型企业合作，积极开发城市乡村闲置地，建设特色苗圃和观光采摘园，发展林果产业，壮大生态经济。提升罕山、大青沟等5个生物多样性示范区生态服务功能，申报罕山、乌旦塔拉国家级森林公园和大青沟国家级地质公园，保护建设阿古拉、孟家段湿地公园和自然保护区，沿科尔沁500公里风景大道实施绿色长廊工程。全面加强森林草原火灾和有害生物防控，确保生态安全。

整治环境突出问题。深入开展大气、水、土壤污染防治行动，解决重点区域、重点行业、重点企业环境隐患问题，全力做好中央环保督察反馈意见的整改落实工作。完善重污染天气预警会商机制和区域联防联控机制，扎实推进北部煤电铝烟气污染物趋零排放示范工程等重点环保项目建设。加强工业园区、农牧业面源污染和矿山地质环境综合治理。强化环境监管执法网格化管理，加大环境执法监管力度，严肃查处环境违法行为。开展节能降耗技术改造，实施重点排污企业在线、工况、视频“三位一体”自动监控，保证主要污染物排放稳定下降、环境质量稳步提高。

加强生态文明制度建设。全面落实主体功能区规划，划定生态保护红线、永久基本农田、城镇开发边界，明确城镇、农业、生态空间发展布局，实行重点生态功能区产业准入负面清单制度。实施最严格的水资源管理制度，建设节水型社会，确保饮用水水源地安全达标。认真落实禁牧休牧、草畜平衡制度，持续开展“三滥”治理，巩固扩大生态建设成果。继续实施领导干部自然资源资产离任审计试点。完成国有林场改革，完善集体林权制度。实施全民节能行动计划，积极培育生态文化，倡导绿色低碳生活方式，形成崇尚生态文明、共促绿色发展的社会风尚。

（九）着力增进各族人民福祉

民生是我们一切工作的出发点和落脚点，人民的期盼就是我们的努力方向。

强力推进“脱贫攻坚提质工程”。 把脱贫攻坚作为重大政治任务、第一民生工程和最大发展机遇。严格落实脱贫攻坚责任，实施最严格的评估考核，严肃查处假脱贫、“被脱贫”、数字脱贫，确保脱贫得到群众认可、经得起历史检验。建立完善精准扶贫大数据管理平台，精准识别贫困人口，逐户逐人实施精准脱贫，确保不落一户、不漏一人。加大脱贫攻坚投入力度，整合投入各类资金45亿元，通过发展产业、易地搬迁、生态补偿、教育扶贫、政策兜底等措施，精准扶持3.5万贫困人口稳定脱贫，开鲁县、扎鲁特旗实现脱贫摘帽。大力推进产业扶贫，加大金融扶贫力度，加快发展肉牛、特色种植、庭院经济、乡村旅游、农村电商和光伏等脱贫产业，力争贫困人口年人均收入增幅超过15%。针对因病致贫比例偏高实际，将建档立卡贫困人口住院就医和大病保险报销起付线降低50%，报销比例提高5个百分点，通过大病保险、商业补充保险等方式，弥补高额医疗支出费用。实行先诊疗后付费、家庭病床、民政救助等综合措施，确保患病贫困群众及时入院、尽早治疗，切实解决因病致贫、因病返贫问题。加大教育扶贫力度，制定教育扶贫政策措施，对建档立卡贫困家庭的小学、初中、高中、中职在校生给予定额补助，将低保家庭大学生每人每年1万元资助政策覆盖到所有建档立卡贫困家庭，不让一个学生因贫失学、一个家庭因学返贫，使更多孩子成就梦想、更多家庭实现希望。

提高基本民生保障能力。实施就业优先战略，分类做好重点群体就业，力争实现城镇新增就业1.7万人，确保零就业家庭动态归零。全面落实促进城乡居民增收的各项政策措施，确保城乡居民收入稳定增长。着力促进农牧民收入较快增长，增加经营性收入和政策性收入，扩大财产性收入和工资性收入，努力缩小城乡居民收入差距。继续提高退休人员基本养老金。推行全民参保计划，实现法定人员全覆盖。提高城乡居民医保财政补助标准，实现自治区内异地就医住院费用直接结算。执行地区最低工资标准，扩大企业工资集体协商覆盖面，积极构建和谐劳动关系，维护劳动者合法权益。切实解决好农民工工资拖欠问题。稳步提高优抚、社会救助标准，实施好临时救助制度。加强农村牧区留守儿童关爱保护和城乡困境儿童保障。关心帮助孤寡老人。全面落实残疾人“两项补贴”制度。建立基本生活保障协调机制，切实做好托底工作，使困难群众心里有温暖、生活有奔头。

加快发展各项社会事业。全面落实党的民族政策，深入开展民族团结进步教育和创建活动，保护和发展少数民族优秀传统文化，扎实做好城市民族工作，筑牢各族干部群众“两个共同”“三个离不开”“五个认同”思想根基，创建全国民族团结进步示范市。促进文化繁荣发展。设立科尔沁文化政府奖，支持文化精品创作和科尔沁非物质文化遗产保护传承。编撰《科尔沁文化系列丛书》，建成乌力格尔艺术馆、版画艺术中心，承办第22届全国版画展、全国版画大赛，举办全区“科尔沁杯”乌力格尔大赛。高质量推出《畅想科尔沁》音乐会，逐步打造民族音乐文化高地。建设完成应急广播信号传输、广播电视综合监管平台等广电工程。推进全民阅读工作。优先发展民族教育，实施通辽蒙古族中学改扩建，完成农村牧区中小学“大通铺”改造和教师周转宿舍建设工程，增加中心城区幼儿园数量，对农村牧区小学、教学点进行合理布局，义务教育均衡发展未达标旗县全部通过国家验收。提升职业教育办学水平，提高职普比例。加强校长和教师队伍建设，规范办学行为，提高教育教学质量。启动健康城市创建工作，全面提高人民健康水平。与北京12家三甲医院在远程会诊等方面开展深度合作，方便群众就地就近享受高端医疗服务。完善医疗急救区域网，加快建设全市医疗急救圈。继续采取以奖代补方式加强苏木乡镇中心卫生院能力建设，减轻农牧民就医负担。大力发展蒙中医事业，引进培养蒙中医药人才。提升生育医疗保健服务水平。加快全民健身中心建设，广泛开展全民健身活动，举办首届市民运动大会。巩固发展足球改革成果，办好“通辽杯”足球赛等品牌赛事。做好老龄、维护妇女和未成年人权益工作，大力发展残疾人事业和红十字等慈善公益事业，依法管理宗教事务，发挥好工会、共青团、妇联等人民团体的桥梁纽带作用。

维护社会和谐稳定。深化平安通辽建设，加强社会治理创新，提高预测预警预防各类风险能力。完善立体化社会治安防控体系，大力整治社会治安突出问题，侦破积案，重案必破，增强人民群众安全感。优化服务管理平台，深化基层平安创建，夯实维护社会稳定的基层基础。全力做好信访工作，妥善解决一批重点信访积案。落实重大决策社会稳定风险评估制度，健全社会利益表达、利益协调、利益保护机制，有效预防和化解矛盾纠纷。健全完善公共安全体系，强化安全红线意识，严格落实安全生产责任制，牢固树立“隐患就是事故、事故必须处理”的理念，从严从细从实抓好十大行业领域的安全生产大检查，以零容忍的态度抓好隐患整改，严厉打击各类非法违法生产经营行为，确保不发生重特大安全生产事故。健全突发事件应急处置机制，增强防灾减灾能力，切实维护人民群众生命财产安全。坚持“四个最严”，完善食品药品安全监管体系，保障人民群众饮食用药安全，创建国家食品药品安全市。深入抓好人防工作。实施“七五”普法规划，增强全社会法制意识。深化双拥共建，促进经济建设和国防建设兼容发展，鼓励先进军工技术成果转化落地，推动军民融合深度发展。

三、加强政府自身建设

做好今年工作，加强政府自身建设是保障。各级政府作为“作战部”，要切实增强“四个意识”特别是核心意识、看齐意识，把维护习近平总书记的核心地位当作最大的政治，当作最重要的政治纪律和政治规矩，更加自觉地在思想上政治上行动上同党中央保持高度一致，以强烈的政治责任感和历史使命感，高效能抓好执行落实，确保中央、自治区和市委各项决策部署落地生根、开花结果。

履职尽责，敢于担当。有权必有责，有责要担当。对上级的决策部署要谋在新处、干在实处，坚持规划当先、科学干事，善于把“新蓝图”变成“路线图”和“施工图”，让各项工作由“纸上”落到“地上”，从构想变为现实。要拧紧责任螺丝，对重点工作重点项目要明确责任人、工作要求、成果形式和时间进度，建立全程监管、全面覆盖的工作链条，务求事事有着落、件件有结果；要上紧督查发条，创新督查方式，加大督查问责力度，建立问题及时解决反馈机制，完善绩效考核评价体系，务必把难事做稳妥、小事做精致、大事做圆满；要健全激励机制和容错纠错机制，给干事者鼓劲，为担当者撑腰，努力建设务实担当的政府。

依法行政，讲求诚信。有权不可任性。各级政府要牢固树立法治信仰和法治思维，尊崇法治、敬畏法律、依法办事。政府主要领导要认真履行推进法治建设第一责任人职责，严格依法行政，执行“三重一大”决策制度，加大审计监督力度，让政府的一切权力在法治轨道上运行。认真执行人大及其常委会的决定，主动接受人民政协的民主监督和社会舆论监督。全面推进政务公开，完善新闻发布制度，消除“信息孤岛”，及时回应社会关切。大力加强政务诚信建设，言必信、行必果，以政府诚信引领社会诚信，努力建设法治诚信的政府。

廉洁自律，干净干事。公生明，廉生威。我们要永葆共产党人清正廉洁的政治本色，严格遵守党规党纪，严格落实从严治党主体责任，全面落实党风廉政建设“一岗双责”，坚决守住廉洁从政这条生命线。各级政府要始终把规矩和纪律挺在前面，严格按政策制度办事，按规矩程序办事，持廉守正，不失职、不越权。严格执行中央八项规定精神和自治区党委、市委配套规定，驰而不息反“四风”，坚决整治失职渎职和庸政懒政怠政行为，让廉洁自律成为干部的自觉追求，让干净干事成为政府的作风导向，努力建设清正廉洁的政府。

今年是自治区成立70周年，我们要精心组织好各项迎庆活动，充分展示通辽发展成果和美好前景，动员全市各族干部群众团结一心、奋力拼搏，以优异的成绩向自治区成立70周年献礼！

各位代表，只要努力奋斗，梦想就会成真。让我们更加紧密地团结在以习近平同志为核心的党中央周围，在自治区党委、政府和市委的坚强领导下，发扬一往无前的蒙古马精神和一抓到底的钉钉子精神，不忘初心，继续前进，撸起袖子加油干，谱写通辽跨越发展新篇章，为打造祖国北疆亮丽风景线作出新的更大贡献。
